# Bronchopneumonia Due to *Pasteurella Multocida* (*Septica*)

**Published:** 1961-10

**Authors:** J. A. Hayes, G. Neale

**Affiliations:** The Departments of Clinical Pathology and Medicine, Bristol Royal Infirmary; The Departments of Clinical Pathology and Medicine, Bristol Royal Infirmary


### BRONCHOPNEUMONIA DUE TO *PASTEURELLA MULTOCIDA* (*SEPTICA*)

BY

J. A. HAYES and G. NEALE

*The Departments of Clinical Pathology and Medicine, Bristol Royal Infirmary*

*Pasteurella multocida* (*septica*) is frequently found in domestic animals such as cats and dogs, usually in the mouth or on the paw, and produces no disease. Infection of humans by this organism is uncommon and usually follows a cat-scratch or dog-bite, the lesion being a chronic pyogenic inflammation of the subcutaneous and adjoining structures. The following report is of an attack of bronchopneumonia caused by this organism which resolved after antibiotic therapy.

### CLINICAL HISTORY

A 68-year-old female who lived in the country near Iron Acton, Gloucestershire, was admitted to the Bristol Royal Infirmay on 16th August 1960, complaining of tiredness and breathlessness. She had had occasional indigestion for several years and three days before admission she had vomited blood and passed bulky black stools.

*On examination* she was thin and pale and her finger nails showed early koilonychia. Pulse 128/min. regular rhythm. Blood presusre 120/60. Haemoglobin 21 per cent, reticulocytes 3·4 per cent, leucocytes 8000/per cm. The blood film showed iron deficiency changes. Her abdomen was normal.

Ausculation of the heart showed a gallop rhythm with a soft pericardial rub at the base. An electrocardiogram showed evidence of a recent posterior myocardial infarct.

She had had a slight cough for three weeks with the production of a small amount of mucopurulent sputum. Clinically her lungs appeared normal but an X-ray showed widespread ill-defined opacities throughout both lung fields, an appearance consistent with bronchopneumonia.

### *Treatment*

Because of her bleeding ulcer she was given a blood transfusion and put on an ulcer diet. There was no recurrence of the haematemesis and she had no further indigestion while in hospital. The myocardial infarct showed normal evolution of the electrocardiogram changes associated with healing.

Tetracycline 500 mg six hourly was given for 5 days together with postural drainage and chest percussion, and with this treatment the bronchopneumonia cleared clinically and radiologically. The second X-ray did however show some linear streaking in the right lower lobe suggestive of bronchiectasis.

### *Bacteriology*

The whitish, mucoid sputum was plated on blood and nutrient agar, both aerobically and anaerobically. It gave a profuse growth of a small, Gram-negative coccobacillus which showed appreciable pleomorphism and a varying degree of bipolar staining. It was thought to be of the *Pasteurella* group. The colonies were large (an average diameter of 0.5 cm, the largest 1·0 cm), opaque and convex with entire blue-grey margins and grey-white raised central areas. They were extremely mucoid and closely resembled capsulated coliform bacilli. No capsule was demonstrated. After subculturing three times the colonies were smaller and less mucoid but still did not show the typical colonial appearance of *Pasteurella*. Mobility was not demonstrated at 27°C. or 37°C.

*Biochemical Reactions*

1. Specific for *Pasteurella multocida* (*septica*): Production of acid without gas from glucose, sucrose, mannose and dextrin. Hydrogen sulphide was produced.

2. Differentiating from coliforms and *Klebsiella*: Lactose was not fermented and there was no coagulation of litmus milk. It grew very poorly on McConkie's medium. The methyl red test was negative.

3. Growth on blood agar was slightly inhibited by colonies of *Staphylococcus aureus*. (The reason for this is not known.)

A saline suspension of the organism was not agglutinated by the patient's serum at any dilution up to 1/2048.

The organism was found to be sensitive to chloramphenicol, tetracycline and nitrofurantoin.

0.5 ml of broth culture was inoculated into mice intraperitoneally and these survived. A larger volume was then used and the mice died after two days. *Pasteurellae* were isolated from the blood stream in pure growth, thus indicating a virulent organism, albeit of diminished potency.

### DISCUSSION

Human infections by *Pasteurellae* have been usually associated with cat scratches, dog-bites or with wounding in rural surroundings (Allott *et al.*, 1944, Ericson and Juhlin, 1959, Talbot and Sneath, 1960). Recently reports have been of a more varied type of lesion—appendicitis (Coghlan, 1958), meningitis following a fractured skull base (Hadern, 1938), chronic para-nasal sinusitis (Bartley, 1960) and bronchiectasis (Schipper, 1947, Olsen and Needham, 1952, Cawson and Talbot, 1955). A recent article quoting the *Pasteurella* infections reported by the Public Health Laboratory Service in the period 1957–1959 emphasizes the variety of lesions—96 instances were associated with animal wounding and 51 instances with either chronic bronchitis or bronchiectasis (Williams, 1960).

Our patient had no story of previous chest troubles but the second chest X-ray did suggest that she might have bronchiectasis which would perhaps explain this unusual infection. It is also possible that her resistance to infection was reduced by the anaemia and it is therefore interesting to speculate whether the organism was a commensal in an abnormal bronchial tree or whether it was acquired from one of her many household pets.

A feature of interest is the rapid response of her infection to antibiotic therapy as compared with the usual chronic course especially where there is subcutaneous infection.

A very interesting bacteriological feature was the extremely mucoid colonial appearance which closely resembled that of *Klebsiella pneumoniae* or of a capsulated coliform. This similarity might well lead to the organism being mistakenly identified when present in sputum.

### SUMMARY

This paper describes an attack of bronchopneumonia caused by *Pasteurella multocida* which resolved completely after treatment with tetracycline.

The organism had an unusual mucoid colonial appearance similar to that of a capsulated coliform or of *Klebsiella pneumoniae*.

### BIBLIOGRAPHY

[1] Allott, E. N., Cruickshank, R., Cynlas-Williams, R., Glass, V., Meyer, I. H., Straker, E. A., and Tee, G. (1944). *J. Path. Bact.*, **56**, 411.

[2] Bartley, E. O. (1960). *Lancet*, **ii**, 581.

[3] Cawson, R. A. and Talbot, J. M. (1955). *J. clin. Path.*, **8**, 49.
[4] Coghlan, Joyce D. (1958). *J. Path. Bact.*, **76**, 45.
[5] Ericson, C. and Juhlin, I. (1959). *Acta Path. et Microbiol. Scand.*, **46**, 47.
[6] Hadern, W. (1938) quoted by Cawson and Talbot, *q.v.*
[7] Horne, W. I. and Berlyne, G. M. (1958). *Brit. med. J.*, **ii**, 896.
[8] Olsen, A. M. and Nedham, G. M. (1952). *Am. J. med. Sci.*, **224**, 77.
[9] Schipper, G. J. (1947). *Bull. Johns Hopk. Hosp.* **81**, 333.
[10] Talbot, J. M. and Sneath, P. H. A. (1960). *J., gen. Microbiol.* **22**, 303.
[11] Williams, E. (1960). *Brit. med. J.* **ii**, 1925.

ACKNOWLEDGEMENTS

Our thanks are due to Professor C. B. Perry and Dr. W. A. Gillespie for their help and criticism, and to Dr. Peacock of the Department of Bacteriology, University of Bristol, for performing the mouse virulence test.

